# Supplementary material for: Knowledge and Practices of Digestive Surgeons concerning Specialized Nutritional Support in Cancer Patients: A Survey Study
Source: Nutrients. 2022 Nov 11;14(22):4764. doi: 10.3390/nu14224764 (PMC9698070; doi:10.3390/nu14224764)
Supplement: Supplementary file 1 [file nutrients-14-04764-s001.zip › nutrients-1935181-supplementary.pdf]

Table S1: Study questionnaire

1. What do you consider that is the most appropriate definition of malnutrition?	
☐	Is the nutritional state due to a decrease in the intake or incorporation of nutrients that leads to an alteration n body composition resulting in a reduction of functional and mental capacity, as well as worsening of clinical outcome
☐	Is the one that affects in a very special way to a particular group, such as hospitalized subjects in which disability and disease are common, taking an own entity
☐	Is that in which there is a decrease of albumin and total proteins due to any intercurrent process
☐	None of them

2. In your opinion, with what parameters is malnutrition most accurately diagnosed?	
☐	With the decrease of albumin and prealbumin
☐	With unintentional weight loss together with a fasting or semi-fasting period of more than one week
☐	With a body mass index < 20 kg/m^2
☐	With a decrease of plasma levels of cholesterol and lymphocytes

3. In your opinion, how should be classified malnutrition that occurs in the surgical patient?	
☐	Malnutrition related to the chronic disease with inflammation
☐	Malnutrition related to fasting
☐	Malnutrition related to the acute disease
☐	Protein malnutrition

4. Which of the following questionnaires would you NOT use for nutritional screening?	
☐	MST (Malnutrition Screening Tool)
☐	MUST (Malnutrition Universal Screening Tool)
☐	CHARLSON

☐ MNA-SF (Mini-Nutritional Assessment Short-Form)

5. What definition of enteral nutrition do you consider most appropriate?

☐ Form of feeding in which the physiological route of the access of food to the digestive system is modified by artificial means

☐ Form of feeding in which the physiological route of the access of food to the digestive system is modified by artificial means, or the physicochemical composition of the nutritional mixture

☐ Form of feeding in which the physicochemical composition of the nutritional mixture is modified

☐ None of them

6. In your opinion, where should the tip of a central venous catheter be placed in order to start total parenteral nutrition (TPN)?

☐ In the subclavian vein

☐ In the cephalic vein

☐ In the superior cavoatrial junction

☐ In the brachiocephalic trunk

7. In relation to preoperative nutritional support in patients undergoing a major abdominal surgical procedure, which of the following statements do you consider to be true?

☐ It is indicated in all patients with severe malnutrition for a period of 7-14 days, even if oncological surgery has to be delayed

☐ Surgery should never be delayed to improve the nutritional status if the patient has an oncological disease

☐ It is only indicated in patients who cannot be nourished by the oral route

☐ 7 days of preoperative nutritional support improves the nutritional status (body composition and albumin levels)

8. How long do you consider it is necessary to maintain total parenteral nutrition (TPN) to achieve any postoperative clinical benefit?

☐ At least 3 days

☐ At least 5-7 days

☐ More than 7 days

☐ At least 48 hours

9. What do you consider that are the advantages of enteral infusion of nutrients in the early postoperative period?

Mark all options that apply

☐ To preserve the integrity of the intestinal mucosa (structure and function) and, therefore, the gastrointestinal barrier

☐ To improve blood flow and mesenteric oxygenation

☐ To reduce bacterial translocation and maintain immunocompetence

10. In your opinion, which of the following statements regarding postoperative nutritional support is correct?

☐ TPN should be routinely administered postoperatively in patients with major gastrointestinal surgery

☐ Nutritional support after surgery should be administered in those patients in whom fasting times of at least 3-5 days are anticipated

☐ TPN is indicated postoperative when complications occur in association with intestinal failure, which make it foreseeable that the patient will be unable to meet their nutritional requirements through oral (enteral) intake for a period of 7-10 days in case of being malnourished

☐ TPN is indicated postoperative when complications occur in association with intestinal failure, which make it foreseeable that the patient will be unable to meet their nutritional requirements through oral (enteral) intake for a period of 7-10 days if he/she is normally nourished

11. In most patients, when do you consider that oral diet should be started after major abdominal surgery?

☐ In the first 24 hours

☐ When there is passage of feces

☐ When there is no leak in the intestinal transit

☐ After 5 days because it is considered to be the most appropriate period for the protection of sutures

12. Regarding immunonutrition, add those statements that in your opinion are correct.

Mark all options that apply

☐ Currently there is no clear evidence to recommend its use only in the preoperative period

☐ Its administration is recommended in the peri and postoperative period of malnourished patients with cancer

- ☐ Its use is associated with a significant reduction of postoperative complications and length of hospital stay
- ☐ It is not cost-effective

13. In relation to peripheral parenteral nutrition, select those statements that in your opinion are correct. Mark all options that apply

- ☐ Nutrients are infused into the bloodstream through a peripheral vein
- ☐ With this type of nutritional support, 100% of the patient's nutritional requirements are not usually covered
- ☐ It is indicated for periods of less than 7 days
- ☐ It is indicated in those patients who require fluid restriction, e.g. heart or liver failure

14. In a patient presenting with intestinal occlusion requiring a surgical operation, when would you indicate preoperative TPN?

- ☐ Always, regardless of whether he/she is going to be operated in the next 48 hours
- ☐ Only in patients with mild malnutrition and for a period of at least 5 days
- ☐ Only in patients with severe malnutrition and for a period of at least 7-10 days
- ☐ In all patients regardless their nutritional status for at least 7 days

15. In a patient with carcinoma of the esophagus without preoperative weight loss who will undergo esophagectomy, which of the following attitudes do you consider that are correct?

- ☐ Is a patient without preoperative malnutrition, so that postoperative nutritional support is not required
- ☐ Because it is expected to maintain the patient in fasting conditions for at least 5-7 postoperative days, it is recommended to administer preoperative nutritional supplements
- ☐ Due to a presumed prolonged lack of postoperative oral intake, it is recommended to administer TPN for at least 7-10 days
- ☐ In any case, enteral nutrition through jejunostomy would be indicated

16. According to the protocol of your hospital, what is the procedure to follow when faced with a patient with cancer of the pancreas and weight loss of 20 kg in the last 3 months and BMI of 18 kg/m^2?

- ☐ Schedule oral supplements from the first visit to the clinic, while the staging study is completed
- ☐ Make a nutritional screening by the own surgeon and to decide whether administration of oral supplements is indicated

- [ ] Refer to the Endocrinology/Nutrition consultation to assess the nutritional status and decide on the appropriate treatment
- [ ] Admit the patient to the hospital for TPN while tumor stating evaluation is being completed

17. When faced with a patient with obstructive and locally advanced gastric cancer, indicate the attitude that seems most appropriate to you

- [ ] An endoscopic endoprosthesis should be placed to allow correct oral feeding during neoadjuvant treatment
- [ ] Curative surgery should be consider as the first step and subsequent adjuvant treatment
- [ ] During staging laparoscopy, jejunostomy should be performed in order to administer enteral nutrition while neoadjuvant treatment is used
- [ ] The patient is candidate for home TPN while receiving neoadjuvant treatment

18. A patient who had undergone laparoscopic right hemicolectomy presents a paralytic ileus during 5 days, what would be the attitude to follow?

- [ ] It is expected that it would be resolved in a short time, so that peripheral parenteral nutrition may be sufficient
- [ ] TPN for at least 5-7 days
- [ ] Once occlusion by a mechanical cause has been excluded, the introduction of oral feeding is the best method for preventing paralytic ileus
- [ ] After a right hemicolectomy, paralytic ileus is frequent, so that preoperative supplementation is indicated even in normonourished patients

19. In a patient with sigmoid rectal cancer you decide to apply an ERAS protocol, which would be your preoperative recommendations?

- [ ] As it is a rectal cancer, mechanical colon preparation, 8-hour preoperative fasting and starting oral feeding at 24 hours after surgery with nutritional supplements
- [ ] No mechanical bowel preparation, 8-hour fasting for solids and 2-hour for liquids, and starting oral fluid intake at 6 hours after surgery
- [ ] Preoperative nutritional optimization using oral supplements up to 8 hours before surgery, no mechanical colon preparation and starting tolerance to fluids by the oral route at 12 hours after surgery
- [ ] Mechanical colon preparation associated with oral vancomycin, 8-hour fasting, and starting oral fluid intake at 6 hours after surgery

20. A patient who underwent laparoscopic right hemicolectomy presents postoperative pain and a morphine pump is prescribed. The patient presents nausea, vomiting and prolonged ileus which delays oral feeding up to 7 days after surgery, which of the following ERAS measures do you think that could have changed the course of the patient?

- [ ] The patient is operated on by laparoscopic surgery and this is the most effective method to reduce pain and postoperative ileus

☐ Multimodal analgesia through a lumbar epidural catheter

☐ Infiltration of the laparoscopic ports or bilateral transversus abdominis plane (TAP) block to reduce the need of rescue opioids

☐ All the above answers are true

21. When faced with an obese patient diagnosed with colon cancer, what would be your attitude in the consultation?

☐ I would indicate a diet because obesity increases the risk of postoperative complications

☐ I would not pay attention to the nutritional status, but I would pay attention to possible anemia, which must be optimized prior to surgery

☐ I would perform a nutritional screening as in all patients

☐ I would prescribe hypocaloric hyperprotein supplements to reduce weight before the intervention, but ensuring an adequate level of proteins
